# Eyebrow Ptosis After Blowout Fracture Indicates Impairment of Trigeminal Proprioceptive Evocation That Induces Reflex Contraction of the Frontalis Muscle

**Published:** 2013-06-20

**Authors:** Ryokuya Ban, Kiyoshi Matsuo, Midori Ban, Shunsuke Yuzuriha

**Affiliations:** Department of Plastic and Reconstructive Surgery, Shinshu University School of Medicine, Matsumoto, Japan

## Abstract

**Objective:** The mixed levator and frontalis muscles lack the interior muscle spindles normally required to induce involuntary contraction of their slow-twitch fibers. To involuntarily move the eyelid and eyebrow, voluntary contraction of the levator nonskeletal fast-twitch muscle fibers stretches the mechanoreceptors in Müller's muscle to evoke trigeminal proprioception, which then induces reflex contraction of the levator and frontalis skeletal slow-twitch muscle fibers. The trigeminal proprioceptive nerve has a long intraorbital course from the mechanoreceptors in Müller's muscle to the superior orbital fissure. Since external force to the globe may cause impairment of trigeminal proprioceptive evocation, we confirmed how unilateral blowout fracture due to a hydraulic mechanism affects ipsilateral eyebrow movement as compared with unilateral zygomatic fracture. **Methods:** In 16 unilateral blowout fracture patients, eyebrow heights were measured on noninjured and injured sides in primary and 60° upward gaze and statistically compared. Eyebrow heights were also measured in primary gaze in 24 unilateral zygomatic fracture patients and statistically compared. **Results:** In the blowout fracture patients, eyebrow heights on the injured side were significantly smaller than on the noninjured side in both gaze. In the zygomatic fracture patients, eyebrow heights on the injured side were significantly larger than on the noninjured side in primary gaze. **Conclusion:** Since 60° upward gaze did not recover the eyebrow ptosis observed in primary gaze in blowout fracture patients, such ptosis indicated impairment of trigeminal proprioceptive evocation and the presence of a hydraulic mechanism that may require ophthalmic examination.

Since the lower-positioned transverse ligament develops in the lowest fat pad space and antagonizes eyelid retraction that depends on contraction of the levator and Müller muscles in the Japanese, the eyelid-opening muscles consist of the levator, Müller, and frontalis muscles (Fig 1a).^1^^,^^2^ Although the frontalis muscle consists of skeletal fast-twitch and slow-twitch muscle fibers, it lacks the interior muscle spindles normally required to induce reflex contraction of the slow-twitch fibers.^3^ Because of bilateral corticofacial innervation, voluntary contraction of the bilateral frontalis fast-twitch fibers symmetrically raises the eyebrows with the anterior lamella of the upper eyelid.^4^ The levator muscle consists of nonskeletal fast-twitch fibers that embryologically derive from fibers in the global layer of the superior rectus muscle^2^ and skeletal slow-twitch fibers which lack interior muscle spindles in the same manner as the frontalis slow-twitch fibers (Fig 1a).^3^^,^^6^ The supratarsal Müller's muscle is innervated not only efferently by sparse sympathetic fibers with interstitial cells of Cajal but also afferently by trigeminal proprioceptive fibers in a palisade arrangement of mechanoreceptors to induce reflex contraction of the levator and frontalis skeletal slow-twitch fibers (Figs 1a and 2).^2^,^7^^-^13 The trigeminal proprioceptive nerve fibers converge as a transverse nerve on the proximal Müller's muscle, join into the lacrimal branch of the ophthalmic trigeminal nerve, pass through the superior orbital fissure, and reach the mesencephalic trigeminal nucleus neighboring the locus ceruleus. The trigeminal proprioceptive nerve fibers innervate the levator and frontalis motor neurons for reflex contraction of skeletal slow-twitch fibers (Figs 1a and 2).^2^^,^^6^^,^^13^^,^^14^ Transcutaneous electrical stimulation of the transverse trigeminal proprioceptive nerve on the proximal Müller's muscle can provoke monosynaptic short latency responses in the ipsilateral levator and frontalis muscles, which appear to induce phasic movement of the eyelid and eyebrow according to vertical gaze changes.^15^^-^17

To involuntarily and effectively move the eyelid and eyebrow, voluntary contraction of the levator and superior rectus nonskeletal fast-twitch muscle fibers in a degree-dependent manner stretches the mechanoreceptors in Müller's muscle to evoke trigeminal proprioception. According to vertical gaze changes, this involuntarily induces phasic reflex contraction of the levator and frontalis skeletal slow-twitch muscle fibers to maintain an adequate visual field in a kind of length servo mechanism. To gaze upward, increased voluntary contraction of the levator and superior rectus nonskeletal fast-twitch muscle fibers further enhances stretching of the mechanoreceptors in Müller's muscle to involuntarily increase phasic reflex contraction of the levator and frontalis skeletal slow-twitch muscle fibers, resulting in involuntary increases in the eyelid and eyebrow heights.^18^

The trigeminal proprioceptive nerve that conveys the stimulus to induce reflex contraction of the levator and frontalis slow-twitch fibers has a long intraorbital course from the mechanoreceptors in Müller's muscle to the superior orbital fissure.^5^ Because external force to the globe may impair trigeminal proprioceptive evocation, we confirmed how unilateral blowout fracture by means of a hydraulic mechanism affects the ipsilateral eyelid and eyebrow movements as compared with unilateral zygomatic fracture.

## PATIENTS AND METHODS

As model patients whose trigeminal proprioceptive evocation may have been impaired, 16 patients with unilateral orbital floor blowout fracture caused by external forces to the globe (11 men and 5 women, 33.1 ± 17.5 years old) were enrolled in this study (Figs 3a-3h). As model patients whose trigeminal proprioceptive evocation may not have been impaired, 24 patients with unilateral zygomatic fracture (16 men and 8 women, 35.8 ± 17.2 years old) were also enrolled (Figs 4i and 4j). Because all patients with blowout or zygomatic fracture were surgically reduced, diagnosis was confirmed by CT scan and intraoperative observation.

Preoperative vertical palpebral fissure and eyebrow height between the uppermost margin of the eyebrow above the center of the pupil and the intercanthal line were measured on both noninjured and injured sides in primary gaze and, for blowout fracture patients, in 60° upward gaze toward a corresponding target marked on the wall in front of the patients. These were then statistically compared.

Patients were asked to refrain from voluntarily enhanced contraction of the frontalis muscles while gazing. All measurements were based either on a 10-mm square scale (Casmatch, Dai Nippon Printing Co, Ltd, Tokyo, Japan) attached to the face or the corneal horizontal diameter measured after photographing. The study's protocol was approved by our institutional review board for human subjects. All patients were fully informed about the nature of the study and gave their informed written consent for participation. Data were analyzed using Wilcoxon signed rank test with SPSS (IBM Corporation, Somers, NY). A *P* value of less than .05 indicated a statistically significant difference.

## RESULTS

In primary gaze in patients with blowout fracture, mean eyebrow height on the injured side (26.35 mm) was significantly smaller than on the noninjured side (28.10 mm) (*P* = 0.004) (Fig 4a), while in 60° upward gaze, mean eyebrow height on the injured side (29.36 mm) was also significantly smaller than on the noninjured side (31.26 mm) (*P* = 0.001) (Fig 4b). In primary gaze, mean vertical palpebral fissure on the injured side (6.13 mm) was significantly smaller than on the noninjured side (7.84 mm) (*P* < .0001) (Fig 4c), while in 60° upward gaze, mean vertical palpebral fissure on the injured side (8.87 mm) was significantly smaller than on the noninjured side as well (10.69 mm) (*P* = .0001) (Fig 4d).

Conversely, in primary gaze in patients with zygomatic fracture, mean eyebrow height on the injured side (31.6 mm) was significantly larger than on the noninjured side (29.0 mm) (*P* < .0001) (Fig 4e).

## DISCUSSION

**Figure 1:**
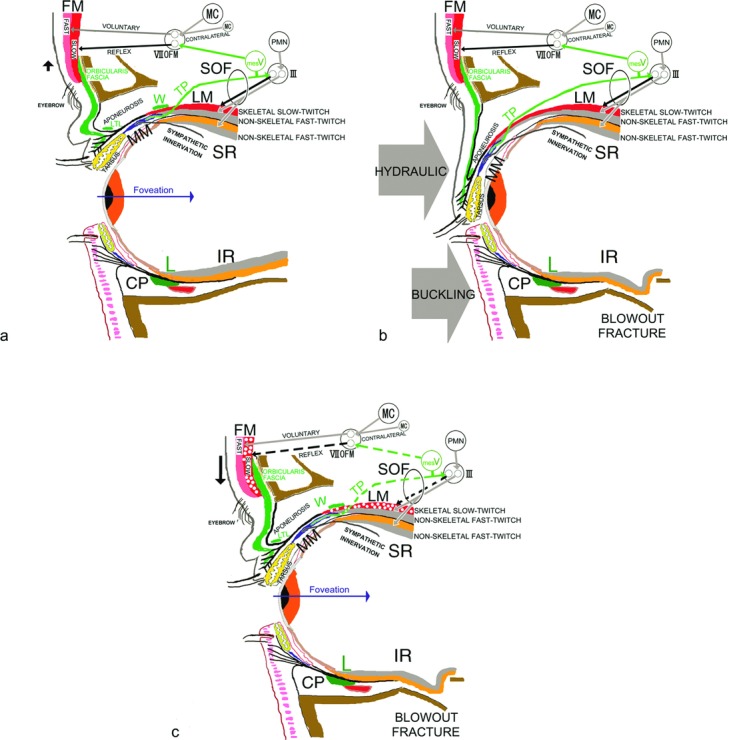
Schematic diagrams of reflex contraction of the levator (LM) and frontalis (FM) skeletal slow-twitch fibers and blowout fracture. (*a*) Normal eyelid. Voluntary contraction of the frontalis skeletal fast-twitch muscle fibers (Fast) is induced by excitation of the ipsilateral or contralateral motor cortex (MC) and the frontalis subnucleus of the facial nucleus (VIIOFM). Voluntary contraction of the levator (LM) and superior rectus (SR) nonskeletal fast-twitch muscle fibers is induced by excitation of premotor neurons (PMN) and oculomotor neurons (III). Voluntary contraction of the levator and superior rectus nonskeletal fast-twitch muscle fibers stretches the mechanoreceptors in Müller muscle (MM) to evoke trigeminal proprioception (TP), which induces reflex contraction of the levator and frontalis skeletal slow-twitch muscle fibers via the mesencephalic trigeminal nucleus (mesV). (*b*) Blowout fracture. A hydraulic injury mechanism is induced by a blow force to the globe, whereas a buckling mechanism is caused by a blow force to the orbital rim. (*c*) After blowout fracture, impairment of trigeminal proprioceptive evocation reduces reflex contraction of the levator and frontalis skeletal slow-twitch muscle fibers, resulting in eyelid and eyebrow ptosis. CP indicates capsulopalpebral fascia; IR, inferior rectus muscle; L, inferior suspensory ligament of Lockwood; LTL, lower-positioned transverse ligament; SOF, superior orbital fissure; W, Whitnall ligament.

**Figure 3:**
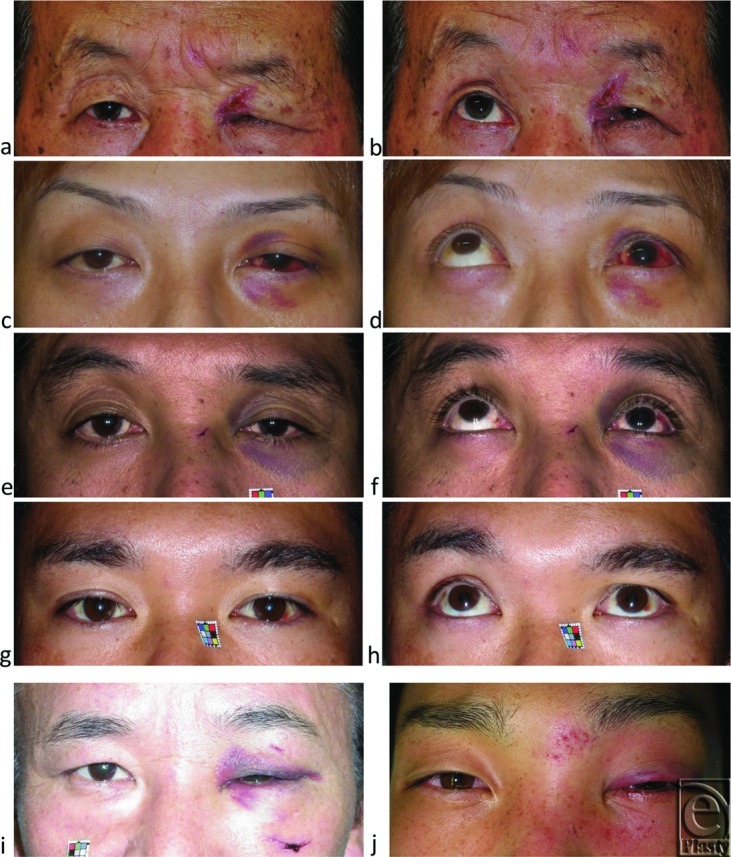
Representative cases of unilateral blowout fractures with eyelid and eyebrow ptosis in primary and 60° upward gaze as well as cases of zygomatic fracture without eyebrow ptosis in primary gaze. (*a*, *b*) Severe eyelid and eyebrow ptosis in a 70-year-old man. (*c*, *d*) Moderate eyelid and eyebrow ptosis in a 32-year-old man. (*e*, *f*) Moderate eyelid and eyebrow ptosis in a 46-year-old man. (*g*, *h*) Eyebrow ptosis in primary gaze was relatively worsened by 60° upward gaze in a 33-year-old man. Severe eyelid ptosis and involuntarily raised eyebrow in 55-year-old (*i*) and 24-year-old (*j*) men with zygomatic fracture.

**Figure 4:**
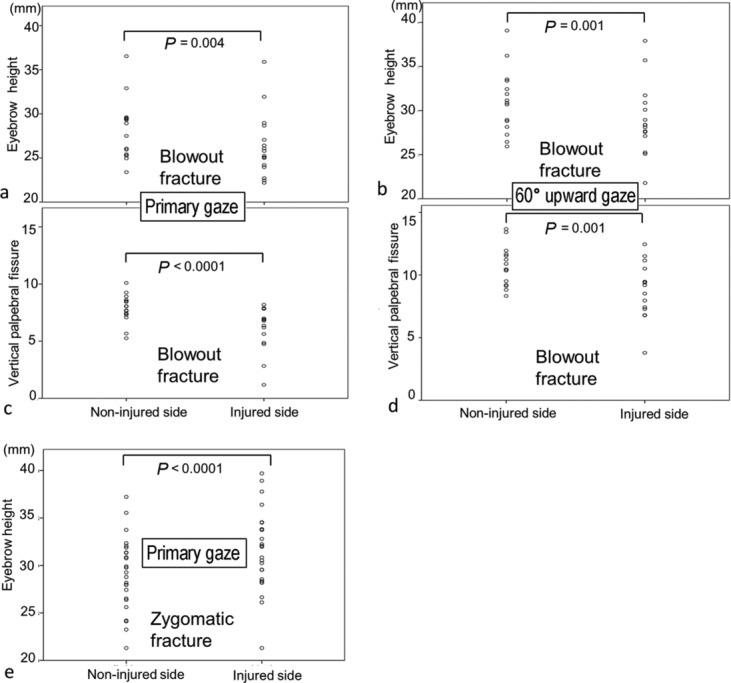
Eyebrow height and vertical palpebral fissure measurements in patients with unilateral blowout or zygomatic fracture in primary and 60° upward gaze. (*a*) Eyebrow heights on noninjured and injured sides in primary gaze in patients with blowout fracture. (*b*) Eyebrow heights on noninjured and injured sides in 60° upward gaze in patients with blowout fracture. (*c*) Vertical palpebral fissures on noninjured and injured sides in primary gaze in patients with blowout fracture. (*d*) Vertical palpebral fissures on noninjured and injured sides in 60° upward gaze in patients with blowout fracture. (*e*) Eyebrow heights on noninjured and injured sides in primary gaze in patients with zygomatic fracture.

In our patients with blowout fracture, both eyelid and eyebrow movements on the injured side were significantly reduced as compared with the noninjured side in both primary and 60° upward gaze. In patients with zygomatic fracture, although eyelid movement appeared to be reduced, eyebrow movement was significantly increased on the injured side. Since the vertical gaze changes to 60° upward gaze in patients with blowout fracture did not recover the relative eyebrow ptosis observed on primary gaze, the eyebrow ptosis on the injured side indicated impairment of trigeminal proprioceptive evocation to induce reflex contraction of the frontalis slow-twitch fibers, even if symmetrical voluntary contraction of the bilateral frontalis fast-twitch fibers was present (Fig 1c).

In response to the motor command to maintain an adequate visual filed in the swollen and ptotic eyelid, increased voluntary contraction of the bilateral levator and superior rectus nonskeletal fast-twitch muscle fibers stretches the mechanoreceptors in Müller's muscle to enhance reflex contraction of the levator and frontalis slow-twitch fibers and increase vertical palpebral fissure and eyebrow height. In patients with zygomatic fracture, because the unilateral loading caused by the swollen eyelid burdens the mechanoreceptors in Müller's muscle to ipsilaterally enhance intact trigeminal proprioceptive evocation, the resulting increased reflex contraction of the ipsilateral levator and frontalis slow-twitch fibers cannot increase vertical palpebral fissure due to eyelid load but increases eyebrow height on the injured side. In patients with blowout fracture whose trigeminal proprioceptive evocation is impaired, the correspondingly decreased reflex contraction of the ipsilateral levator and frontalis slow-twitch fibers cannot increase either vertical palpebral fissure or eyebrow height on the injured side.

There are 3 possibilities as to how trigeminal proprioceptive evocation was impaired in the blowout fracture group. First, the trigeminal proprioceptive nerve fibers between the mechanoreceptor in Müller's muscle and the superior orbital fissure might have been injured, especially in the Müller's muscle facing the blow force (Fig 1b). Second, entrapment of the inferior rectus muscle or the orbital contents surrounding the inferior rectus muscle into the orbital floor might have prevented increased voluntary contraction of the levator and superior rectus nonskeletal fast-twitch muscle fibers from sufficient stretching of the mechanoreceptors in Müller's muscle. Third, because extension of the orbit due to blowout fracture rendered the orbital contents to be inferiorly and posteriorly positioned, the newly displaced levator and superior rectus non-skeletal fast-twitch muscle fibers might have been insufficient to stretch the mechanoreceptors in Müller's muscle and induce reflex contraction of the levator and frontalis slow-twitch muscle fibers.

We believe that injury to trigeminal proprioceptive nerve fibers was the most likely scenario in the blowout fracture by means of the hydraulic mechanism. According to Seddon classification,^19^ peripheral nerve injuries are classified into neuropraxia, axonotmesis, and neurotmesis. Because reflex contraction of the levator and frontalis slow-twitch fibers was preserved on the injured side, blunt trauma to the globe causing blowout fracture was presumed to induce neuropraxia and axonotmesis at most. As neuropraxia is a localized, physiological, and transient conduction block, recovery from eyelid and eyebrow ptosis ranges from days to weeks. Axonotmesis involves axonal degeneration in the intact endoneurial tube, and recovery often spans from weeks to months.

As far as the mechanism of blowout fracture is concerned, a buckling mechanism caused by trauma to the orbital rim and a hydraulic mechanism caused by trauma to the globe through the closed eyelid have both been proposed (Fig 1b).^20^^,^^21^ A blow force to the globe has a greater possibility of causing trigeminal proprioceptive evocation impairment (Fig 1c). Therefore, the presence of eyebrow ptosis on the injured side support the notion of a hydraulic mechanism, for which the patient may require further ophthalmologic examination.

Blunt trauma to the brain increases susceptibility to abducens nerve palsy because cranial nerve VI has the longest subarachnoid course among all cranial nerves.^22^ Similarly, blunt trauma to the globe may raise the likelihood of trigeminal proprioceptive nerve damage because the afferent nerve has the longest intraorbital course among all nerves related to eye and eyelid movements.^6^ However, blunt trauma to the globe is also thought to cause orbital apex syndrome^23^ and superior orbital fissure syndrome^24^ with or without blowout fracture. Orbital apex syndrome involves cranial nerves II, III, IV, V1, and VI, while superior orbital fissure syndrome involves cranial nerves III, IV, V1, and VI. Injury to the optic nerve leads to visual impairment that ranges from partial to complete. Injury to cranial nerves III, IV, and VI leads to extraocular and levator muscle nerve palsy, which manifest as diplopia and eyelid ptosis, respectively, although the presence of diplopia does not enable differentiation between injury to the oculomotor nerve and mechanical entrapment of the extraocular muscle in blowout fracture. Injury to cranial nerve V1 leads to sensory disturbance in areas supplied by lacrimal, frontal, and nasociliary branches of the ophthalmic nerve. Trigeminal sensory input is classified into discriminative touch, proprioception, and pain and temperature. Injury to the trigeminal proprioceptive afferent nerve, which is contained in the lacrimal branch of the ophthalmic nerve,^6^ causes impaired involuntary reflex contraction of the levator and frontalis skeletal slow-twitch muscle fibers that manifests as partial to complete eyebrow and eyelid ptosis.

Injury to the unilateral frontal branch of the facial nerve, which also leads to partial to complete ipsilateral eyebrow ptosis ranging from partial to complete, should be differentially diagnosed in blowout fracture with eyebrow ptosis.^25^ However, the presence of symmetrical voluntary contraction of the frontalis fast-twitch muscle fibers in primary gaze^3^ in the Asian narrow eye and the absence of reflexive contraction of the frontalis slow-twitch muscle fibers in primary gaze in the Caucasian eye^26^ often interfere with the assessment of eyebrow ptosis. Because 60° upward gaze or transcutaneous electrical stimulation of the transverse trigeminal proprioceptive nerve on the proximal Müller's muscle induces reflex contraction of the frontalis muscles, they may be useful to confirm the degree of trigeminal proprioceptive nerve injury in nerve conduction studies.

## CONCLUSIONS

Although it may be difficult to differentiate loaded eyelid ptosis due to traumatic swelling from functional eyelid ptosis caused by reduced reflexive contraction of the levator slow-twitch muscle fibers after blowout fracture, the presence of eyebrow ptosis in primary gaze, which is not recovered by 60° upward gaze, indicates impairment of trigeminal proprioceptive evocation that induces reflex contraction of the levator and frontalis slow-twitch muscle fibers. Ophthalmic observation is recommended in such cases. The next step in this investigation would be to confirm the presence of trigeminal proprioceptive nerve damage.

## Figures and Tables

**Figure 2:**
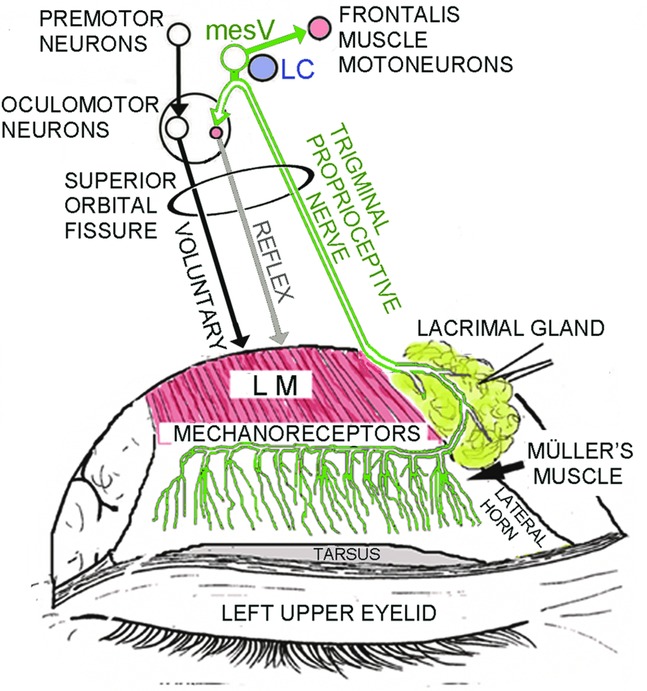
Trigeminal proprioceptive fibers innervating the mechanoreceptors in Müller's muscle that induce reflex contraction of the levator and frontalis slow-twitch muscle fibers. LC indicates locus ceruleus; LM, levator muscle; mesV, mesencephalic trigeminal nucleus.
